# The Humoral Immune Response to BCG Vaccination

**DOI:** 10.3389/fimmu.2019.01317

**Published:** 2019-06-11

**Authors:** Rachel Tanner, Bernardo Villarreal-Ramos, H. Martin Vordermeier, Helen McShane

**Affiliations:** ^1^The Jenner Institute, Nuffield Department of Medicine, University of Oxford, Oxford, United Kingdom; ^2^Department of Bacteriology, Animal and Plant Health Agency, Addlestone, United Kingdom; ^3^Institute of Biological, Environmental and Rural Sciences, Aberystwyth University, Aberystwyth, United Kingdom

**Keywords:** humoral immunity, BCG vaccine, antibodies, B cells, tuberculosis, bovine TB

## Abstract

Bacillus Calmette Guérin (BCG) is the only currently available vaccine against tuberculosis (TB), but it confers incomplete and variable protection against pulmonary TB in humans and bovine TB (bTB) in cattle. Insights into the immune response induced by BCG offer an underexploited opportunity to gain knowledge that may inform the design of a more efficacious vaccine, which is urgently needed to control these major global epidemics. Humoral immunity in TB and bTB has been neglected, but recent studies supporting a role for antibodies in protection against TB has driven a growing interest in determining their relevance to vaccine development. In this manuscript we review what is known about the humoral immune response to BCG vaccination and re-vaccination across species, including evidence for the induction of specific B cells and antibodies; and how these may relate to protection from TB or bTB. We discuss potential explanations for often conflicting findings and consider how factors such as BCG strain, manufacturing methodology and route of administration influence the humoral response. As novel vaccination strategies include BCG prime-boost regimens, the literature regarding off-target immunomodulatory effects of BCG vaccination on non-specific humoral immunity is also reviewed. Overall, reported outcomes to date are inconsistent, but indicate that humoral responses are heterogeneous and may play different roles in different species, populations, or individual hosts. Further study is warranted to determine whether a new TB vaccine could benefit from the targeting of humoral as well as cell-mediated immunity.

## Introduction

Tuberculosis (TB), caused by *Mycobacterium tuberculosis* (*M.tb*), continues to pose a major global health threat with 10 million new cases and 1.6 million deaths per year (1). Bovine TB (bTB), caused mainly by the related *Mycobacterium bovis* (*M.bovis*), is one of the principal diseases reducing livestock production and thus wealth in Africa, South Asia and South America, with an estimated global annual impact of US $3 billion (2, 3). bTB also has public health implications, and in 2017 the World Health Organization (WHO), Food and Agriculture Organization of the United Nations (FAO), World Organization for Animal Health (OIE), and Union against Tuberculosis and Lung Disease published a roadmap to combat zoonotic TB (4). Bacillus Calmette Guérin (BCG) is the only currently available vaccine against TB and potentially bTB, but confers incomplete and variable protection against pulmonary TB in humans and against bTB in cattle (2, 5–7).

Due to the ability of mycobacteria to survive the intracellular environment of phagocytic cells, and the demonstration of specific T cells as the pillar of acquired immunity to TB (8, 9), immunology studies and TB/bTB vaccine development to date have focused on cell-mediated immunity. Indeed, there is ample evidence that the cellular response is necessary for protection, notably the increased susceptibility to TB in mice and humans lacking elements of T cell immunity (10–12). T cell responses to BCG vaccination have been described, indicating the induction of robust Th1 responses in adults and infants (13, 14). However, the association between vaccine-induced cell-mediated immunity and protection against TB is unclear (15, 16). The TB vaccine candidate MVA85A did not demonstrate protective efficacy in a recent Phase IIb trial despite inducing an IFNγ-secreting CD4^+^ T cell response (17). It is not known whether protection requires a more potent cellular response or qualitatively different immunogenicity. A role for unconventional T cells and trained innate immunity in BCG-mediated protection against TB has recently been proposed (18, 19), as well as the potential involvement of humoral immunity (20).

The humoral immune response in the context of TB and bTB has been understudied, but recent evidence suggests that B cells and antibodies may play a more significant role than previously appreciated (20, 21). An antibody response to the Ag85 complex was associated with superior outcome in a cohort of *M.tb*-infected Mexican Indians (22). Furthermore, low titers of antibodies against the mycobacteria surface glycolipid lipoarabinomannan (LAM) have been associated with disseminated TB in children (23), and some evidence suggests that monoclonal antibodies against arabinomannan (AM; the sugar component of LAM) are protective in mouse infection models (24, 25). Lu et al. recently applied a systems serology approach to identify antibody features that distinguished active TB patients from individuals with latent *M.tb* infection (LTBI), indicating a role for antibody effector functions in control of infection (26). Exposed but healthy healthcare workers have also been shown to make antibodies that offer moderate protection against *M.tb* infection in both mouse aerosol challenge and *in vitro* models (27).

The antibody response to BCG vaccination has widely been considered of little relevance to protection (13). However, in a recent *post-hoc* correlates of risk analysis using samples from the MVA85A efficacy trial, levels of Ag85A-specific IgG were associated with reduced risk of TB disease in BCG-vaccinated South African infants (28). Though increased following MVA85A vaccination in the BCG-MVA85A group, levels of these antibodies were also increased in the BCG-only placebo group at this time-point, suggesting that protective antibodies may be induced by BCG vaccination (28). Potential mechanisms of antibody-mediated immunity against *M.tb* include enhanced phagocytosis, increased phagolysosome formation, bacterial neutralization, enhanced inflammasome activation, and enhanced cytotoxic NK cell activity (29). Antibodies may also interact with T cells to promote immunity, for example through forming bacterial-antibody complexes that result in increased processing and presentation of *M.tb* antigens to CD4^+^ T cells by phagocytes, thus increasing T cell activation and enhancing cytotoxic responses (29).

We review what is known about the humoral immune response to BCG vaccination and re-vaccination across species, including evidence for the induction of specific B cells and antibodies and how these may relate to protection from TB or bTB. We consider how factors such as BCG strain, manufacturing methodology and route of administration influence the humoral response, as well as the immunomodulatory effects of BCG vaccination on non-specific humoral immunity. Insights into the BCG-induced specific immune response may aid in informing the design of an efficacious new TB vaccine which could benefit from the targeting of humoral, as well as cell-mediated, immunity. Understanding the off-target effects of BCG is also important in the context of TB vaccine development, as novel candidates in the current pipeline include both homologous and heterologous BCG prime-boost regimens. Understanding the mechanisms of non-specific immunity opens the possibility of replicating or boosting beneficial responses to the host advantage, or conversely of avoiding exacerbation of undesirable effects.

## The B Cell Response to BCG Vaccination

B cells are observed in the lungs of TB patients, in the granulomas of mice and non-human primates (NHPs) infected with *M.tb*, and in cattle infected with *M.bovis*; these cells may act to orchestrate and modulate the immune environment of the infected lung (30–33). There is evidence of a role for B cells in the granuloma producing *M.tb*-specific antibodies, acting as antigen presenting cells (APCs) and reducing immunopathology (32, 34, 35). Humans with pulmonary TB disease have been shown to have lower peripheral B cell counts than asymptomatic individuals with or without *M.tb* infection (36, 37). Vordermeier et al. demonstrated an increased susceptibility to TB in B cell deficient mice, although interestingly specific T cell responses or protection conferred by BCG vaccination were not impaired (38). Bosio et al. also reported that although B cell deficient mice had comparable bacterial loads in the lungs following low-dose infection with an *M.tb* clinical isolate, there was less severe lesion formation and delayed dissemination of bacteria (39). It was later shown that adoptive transfer of B cells into B cell knock-out (KO) mice reversed the enhanced susceptibility to infection and immunopathology observed (40). However, others have reported no role for B cells in such models, which may reflect differences in genetic backgrounds or strain and dose of *M.tb* used for infection (39–42). The B cell compartment may even be involved in disease progression, with an abundance of antibodies in sera of patients with active disease suggesting that B cells are involved in immunopathology (34), although the direction of causality is unclear.

There is a paucity of literature on the B cell response to BCG vaccination (13), although it has been demonstrated in mice that B cell deficiency results in a diminished BCG vaccine-induced Th1 response. Using a series of elegant experiments, the authors proposed that B cells regulate neutrophilia during *M.tb* infection and BCG vaccination by modulating the IL-17 response, and that exuberant early neutrophilia (as observed in B cell deficient mice) can adversely affect the vaccine-induced Th1 response by impairment of dendritic cell (DC) migration to draining lymph nodes, thus compromising CD4^+^ T cell priming (43).

A clinical study of 79 BCG-vaccinated and 14 unvaccinated human volunteers found a significantly higher frequency of PPD-specific memory B cells in PBMC from BCG-vaccinated compared with unvaccinated volunteers (44). A lack of B cell reactivity to either ESAT-6 or CFP-10 indicated that the increased B cell response was not due to *M.tb* exposure. Donors received BCG vaccination between 13 and 45 years prior to enrolment, suggesting a long duration of the memory B cell response (44). Conversely, in a randomized trial of 118 Danish infants, there was limited overall impact of neonatal BCG vaccination on a range of measured B or T cell subset frequencies (45). However, it should be noted that proportions of cells present in the peripheral blood may not correlate linearly with specific immunity, and that effects of antigen-specific cells may be masked where total populations are measured.

## The Specific Antibody Response to BCG Vaccination

### BCG Vaccination in Humans

There have been several studies measuring the specific antibody response to BCG vaccination, a summary of which is provided in Table 1. Early investigations utilized haemagglutination assays; the first serodiagnostic test for TB described by Middlebrook and Dubos in 1948 (78). Lagercrantz and Enell reported a large study of 248 healthy Swedish children aged 8–15 years who were BCG-vaccinated during their first year at school. Sixty-five percent of the children had a haemagglutination titer of at least 1/8; a level comparable to that observed during *M.tb* infection (46). Titer values at 3 months post-vaccination were significantly higher than at 1–5 years post-vaccination, and were higher than those found in children vaccinated at birth (46, 79). While other studies in the 1950s using this assay failed to observe elevated antibody activity following routine BCG vaccination (61), some achieved positive results using alternative methods such as agar gel diffusion and the bentonite flocculation test (47, 80).

**Table 1:**
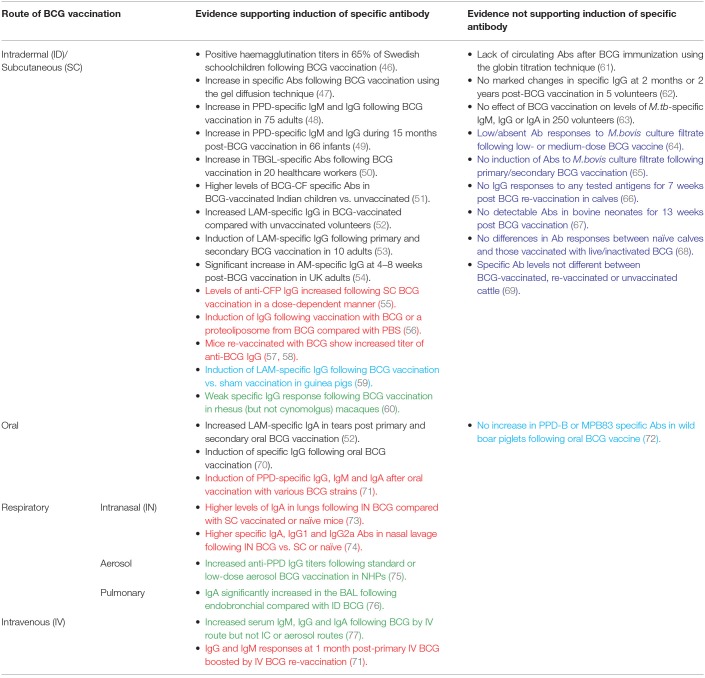
Summary of studies discussed in this review measuring specific antibody responses to BCG by route of administration.

*Human studies are indicated by black text, cattle studies are indicated by blue text, murine studies are indicated by red text and non-human primate studies are indicated by green text. Studies in any other species are indicated by light blue text*.

The 1980s saw the development of an enzyme-linked immunosorbent assay (ELISA) to measure antibody responses in TB using plasma-membrane antigen from *M.tb* (62). In addition to investigating the activity of specific antibody in sera from TB cases, Krambovitis considered the effect of BCG vaccination. Sera were collected from five healthy TST negative adults at baseline and 2 months post-BCG vaccination, and from a further two adults at 2 years post-vaccination. There was no marked change in specific IgG following vaccination in any of the volunteers, although the small sample size is a notable limitation (62). Consistent with this finding, there was no effect of previous BCG vaccination on the levels of *M.tb*-specific IgG, IgM, or IgA by ELISA in a separate study of 250 healthy volunteers (63).

However, later studies have more frequently observed induction of specific antibody following BCG vaccination. Turneer et al. reported a small but significant increase in levels of both IgM and IgG following BCG vaccination in 75 adults using a PPD-specific ELISA (48). In a study of 66 infants in Turkey, half of whom received BCG vaccination in the first month and half in the second month after birth, there was a significant increase in PPD-specific IgM during the 15 month post-vaccination study period with a higher titer at 2 and 4 months post-vaccination in infants who were vaccinated in the second month of life. IgG levels decreased at 2 months post-vaccination (which the authors propose may be due to loss of maternally-transferred antibody) but rose progressively thereafter (49). In a separate study of 50 Indian children aged 1–10 years, there were significantly higher levels of antibody to BCG-CF in BCG-vaccinated compared with unvaccinated children or TB patients, which were directed mostly toward the low molecular weight proteins (51). Nabeshima and colleagues reported a significant increase in antibodies directed against anti-tuberculous glycolipid antigen (TBGL) at 2, 4, and 8 weeks following BCG vaccination in 20 TST-negative Japanese healthcare workers, although antibody levels had reduced by 1 year post-vaccination (50).

Brown et al. went on to investigate antigenic targets of specific antibodies in serum from subjects before and after BCG vaccination and from TB patients, conducting ELISAs with 20 different mycobacterial antigen preparations including recombinant MPT63, MPT64, KatG, MPT51, ESAT-6, MTC28, 14kDa protein, 38kDa protein, CFP-10, TbDP, LAM, and Ag85. Only LAM-reactive IgG was significantly increased among BCG-vaccinated subjects compared with a PPD-negative control group. The proportion of subjects with positive LAM responses in the BCG-vaccinated group was similar to that in the active TB group (52). Given that LAM is a major component of the mycobacterial cell wall and has several immunosuppressive effects that favor mycobacterial survival in the host (including downregulation of DC function through interaction with the DC-SIGN receptor), it follows that neutralizing antibodies to LAM or its component AM may contribute to host defense against mycobacterial infection (81, 82). Indeed, studies have now indicated a protective role for monoclonal antibodies against AM, and partial protection conferred by antibodies induced by vaccination with AM-protein conjugates (25, 83). Furthermore, an inverse correlation between the titer of LAM-specific antibodies and risk of disseminated disease has been reported in humans (23).

De Vallière et al. also reported significant induction of LAM-specific IgG following both primary and secondary BCG vaccination in 10 healthy volunteers (53). To our knowledge, this is one of only two studies to have investigated the functional mechanism of action of BCG-induced antibodies. Using GFP-expressing BCG, it was demonstrated that internalization of BCG by neutrophils and monocytes/macrophages was significantly enhanced using post-vaccination compared with pre-vaccination serum (53). Furthermore, the inhibitory effects of these phagocytic cells on mycobacterial growth were significantly enhanced by BCG-induced antibodies; an effect which was reversed by preabsorption of IgG. Vaccine-induced antibodies also enhanced proliferation and IFN-γ production in mycobacterium-specific CD4^+^ and CD8^+^ T cells, highlighting interdependence between the humoral and cellular arms (53). Similarly, Chen et al. observed a significant increase in AM-specific IgG at 4–8 weeks post-BCG vaccination which was able to opsonize BCG and *M.tb*. When BCG was opsonized with post-vaccination sera, there was significant enhancement of phagocytosis, phagolysosomal fusion and intracellular growth inhibition. Reduction of BCG growth in THP-1 cells correlated significantly with post-vaccination AM-specific IgG, suggesting a role for an FcγR-mediated effect (54). Such evidence supports a protective function for BCG-induced antibodies, as summarized in Table 2.

**Table 2:**
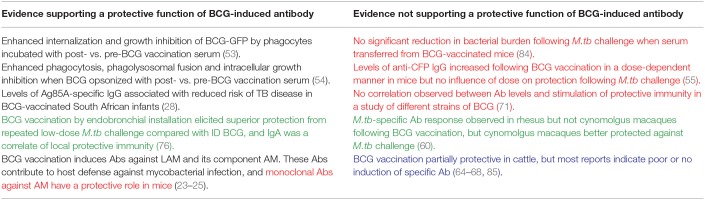
Summary of studies discussed in this review providing evidence for or against a protective function of BCG-induced antibodies.

*Human studies are indicated by black text, cattle studies are indicated by blue text, murine studies are indicated by red text and non-human primate studies are indicated by green text*.

### BCG Vaccination in Cattle

Induction of specific antibody following BCG vaccination is rarely observed in cattle, despite BCG being similarly partially protective in this species. Buddle et al. vaccinated 20 calves with BCG within 8 h of birth, 10 of which received a BCG boost at 6 weeks of age. A further 10 calves received BCG vaccination at 6 weeks only, and 10 matched animals were unvaccinated controls (65). There was no induction of antibody to *M.bovis* culture filtrate in any group at 2, 4, 6, 9, or 12 weeks of age. There was, however, a significant increase in antibody levels in all of the animals following challenge with *M.bovis* (65). Additional studies by the same authors reported that antibody responses to *M.bovis* culture filtrate were low or absent in all groups following low- and medium-dose BCG vaccination and subsequent challenge with *M.bovis*, and following BCG vaccination by the respiratory or subcutaneous (SC) routes (64, 85).

Lyashchenko et al. described a study of six calves aged 6 months that received two immunisations with BCG Pasteur 6 weeks apart. No IgG responses were detected to any of the antigens tested (ESAT-6, CFP-10, MPB70, MBP59, MPB64, MPB83, Acr1, or PstS-1) for 7 weeks post-booster vaccination (66). Furthermore, in a study of 12 healthy bovine neonates aged under 1 month in Ethiopia, there was no detectable antibody in the naïve or BCG-vaccinated groups for 13 weeks post-vaccination (67). In a more recent report of 24 healthy mixed-breed *Bos taurus* calves aged 4–6 months of age, animals were vaccinated with either live or inactivated BCG. There were no significant differences in the antibody responses by IDEXX TB ELISA between naïve animals and those vaccinated with live or inactivated BCG for 9 weeks post-vaccination (68).

It may be of importance that all of these studies were conducted in young calves. When LAM-specific IgG responses to primary and secondary BCG vaccination were measured in calves and young adults, the young adults demonstrated a significant increase in antibody at 9 and 11 weeks post primary vaccination and 1 and 3 weeks after secondary vaccination, while such a response was absent in the calves. Furthermore, PPD-B stimulated cells from vaccinated young adults secreted significantly more antibody than cells from unvaccinated calves in a cell culture system (86). This suggests that young calves may be limited in their capacity to mount an antibody response to BCG, possibly associated with immaturity of the neonatal immune system (87). Furthermore, the passive transfer of maternal antibody to neonates may mask vaccine responses or actively interfere with postnatal activation of the calf's own immune system and capacity to mount a protective response to vaccination or infection (87–89).

### BCG Vaccination in Preclinical Models

Preclinical models offer the opportunity to explore novel vaccine candidates or regimens, and to relate induced immune responses to protection from subsequent experimental mycobacterial challenge. In a murine study comparing different doses of BCG, levels of anti-CFP IgG increased following vaccination at all doses, with the highest levels seen in mice vaccinated with the highest dose of BCG, although there was no influence of dose on protection following *M.tb* challenge (55). A recent murine study of an AM-protein conjugate vaccine demonstrated induction of an AM-specific antibody response, prolonged survival and enhanced control of extrapulmonary dissemination in immunized compared with control animals (84). Interestingly, a serum passive transfer experiment resulted in a significant reduction in lung and spleen bacterial burden following transfer of serum from mice vaccinated with the AM conjugate vaccine constructs but not from mice vaccinated with BCG (84). In a study of mice vaccinated with either PBS, BCG, a proteoliposome from BCG (PLBCG) or PLBCG with alum hydroxide (PLBCG-AL), there was significant induction of specific IgG (all subclasses combined) and IgG1 in all groups receiving vaccines compared with PBS, and significant induction of IgG2a in the BCG-vaccinated group only at 6 weeks post-vaccination (56).

Guinea pigs are a useful model for TB due to ability to reproducibly infect animals with *M.tb* and remarkable similarities to humans in the course of disease following pulmonary infection (90). Consistent with findings in humans, significant induction of LAM-specific IgG has been reported in guinea pigs who had been BCG-vaccinated compared with those receiving a sham vaccination (91). Romain et al. collected sera from BCG-vaccinated guinea pigs and used it to detect mycobacterial antigens present in BCG or *M.tb* culture medium filtrate which are recognized by antibodies raised against live BCG. They identified and purified a complex of 45- and 47-kDa major molecules (Apa), a cell-surface adhesion and secretory glycoprotein produced by all members of the *M.tb* complex, which was found to be an immunodominant target triggering an antibody response following BCG vaccination (59). Several Apa-subunit or DNA vaccines have since been shown to confer significant protection against *M.tb* challenge, consistent across vaccination strategies and animal models (92–96).

While non-human primates (NHPs) are considered the most relevant animal model for human TB vaccine development due to susceptibility to *M.tb* infection and partial protectiveness of BCG (97, 98), there is very little literature on the antibody response to BCG in this model. An early study described detection of antibody in monkeys following BCG vaccination or *M.tb* infection using a gel double-diffusion test (47). In a more recent comparison of BCG vaccination in different species, a weak *M.tb*-specific IgG response was observed in rhesus but not cynomolgus macaques at 14 weeks post-BCG vaccination (although it should be noted that animals were treated with a 3-week course of daily oral isoniazid and rifadin treatment at 8 weeks post-vaccination). Interestingly, cynomolgus macaques appeared better protected against subsequent *M.tb* challenge, which draws into question the relevance of the specific IgG response to protection (60). An increase in anti-PPD antibody titers following aerosol BCG vaccination in macaques has recently been reported, as described in the section Aerosol BCG (75).

### BCG Re-vaccination

Several human epidemiological studies, as well as a large randomized controlled trial in Brazil, have previously indicated that BCG re-vaccination confers no additional protection to neonatal vaccination (99–101), as well as a large randomized controlled trial in Brazil (100). However, a recent Phase II prevention-of-infection trial in South African adolescents found that while BCG re-vaccination did not demonstrate efficacy in preventing initial *M.tb* infection (defined as QFT conversion at an IFN-γ level of ≥0.35 IU per ml after day 84), it did result in significantly reduced rates of sustained QFT conversion (defined as three consecutive positive QFT results after day 84) (102). The authors suggest that as initial acquisition of infection is not averted by the innate immune response, antigen is trafficked to the draining lymph nodes triggering adaptive immunity (as indicated by initial QFT conversion), followed by enhanced bacterial control or clearance in protected individuals (102). The inconsistency in outcomes between this and previous BCG re-vaccination trials may reflect different enrolment criteria, as the recent study excluded adolescents who were not QFT-negative at baseline and BCG efficacy is thought to be greatest in individuals without previous mycobacterial exposure (103). These findings have fuelled renewed interest in the potential utility of BCG re-vaccination, although it is not clear to what extent cellular and/or humoral immunity contribute to this protection.

In the murine model, mice vaccinated with a repeat dose of BCG demonstrated an increased titer of anti-BCG IgG (57, 58). In a study of naïve and historically BCG-vaccinated UK volunteers, there was no significant difference between groups in the total IgG response at baseline or 4 weeks after BCG vaccination (which represented a re-vaccination for the historically BCG-vaccinated individuals). However, the group receiving a re-vaccination had significantly higher levels of IgG2 at both time-points (54). Increases in AM-specific IgA and IgM titers were significant after secondary but not primary vaccination, and re-vaccination induced more pronounced IgG responses to AM oligosaccharide (OS) epitopes than primary vaccination (54). In the de Vallière et al. study described in the section BCG Vaccination in Humans, 8 of the 10 volunteers were given a BCG re-vaccination 6 months following primary vaccination. There was a significant increase in levels of LAM-specific IgG following primary vaccination which was further increased at 8 and 28 weeks post-boost. As described, these antibodies were found to be capable of enhancing both innate and cell-mediated immune responses to mycobacteria (53).

Use of an *in vitro* model of human PBMC induced with a repeat dose of BCG at 24 and 72 h of cell culture demonstrated increased anti-BCG IgG levels in supernatants compared to the pre-boost and control groups (104). Re-vaccination of cattle with BCG 2 years after first vaccination restored protection from *M.bovis* challenge compared with calves receiving only a single vaccination 2.5 years previously (69). However, the serum antibody responses to *M.bovis* culture filtrate protein or Ag85A peptides measured following re-vaccination and challenge were not significantly different between the BCG-vaccinated, re-vaccinated or unvaccinated groups. Interestingly, immune responses in cattle re-vaccinated with TB protein vaccines were biased toward induction of antibody, but this was not associated with improved protection from *in vivo* challenge (69).

## BCG by Other Routes of Administration

### Oral BCG

BCG was originally administered by the oral route, but this was largely replaced by intradermal vaccination following (a) the observation that oral administration produced no allergic skin response (considered at the time evidence of immunity against TB), (b) the Lübeck disaster (when neonates were mistakenly given oral BCG vaccine contaminated with *M.tb*) and (c) association with cervical adenitis (105, 106). However, oral BCG has been shown to induce greater mucosal and systemic immune responses compared with intradermal vaccination in mice (107), and there has been renewed interest in this route of delivery (106). Lagranderie et al. reported the induction of PPD-specific IgG, IgM, and IgA in sera, intestinal secretions and BAL fluids of mice following oral vaccination with various BCG strains (71).

In the Brown et al. study described in the section BCG Vaccination in Humans (52), mucosal specimens were harvested from 8 volunteers before and after two oral BCG vaccinations administered at an interval of 6 months. There was a significant increase in LAM-specific (but not Ag85- or *M.tb* whole lysate-specific) IgA antibodies in tears at 6 months post-primary oral BCG vaccination and at 1 week and 6 weeks post-secondary oral BCG vaccination (52). A second study reported induction of specific IgG that peaked between 10 and 14 days after oral BCG vaccination. Two volunteers who received boosting with oral BCG showed an alteration in the humoral immune response with a shift of isotype from IgG to IgA, independent of the route used for primary vaccination (70). This supports the suggestion by Hoft et al. that a combination of intradermal and oral routes for BCG vaccination may induce mucosal and systemic immunity against initial infection and systemic progression (108).

Significant wildlife reservoir hosts exist for *M.bovis* infection of cattle including badgers in the UK, white-tailed deer in the US, and wild boar and red deer in Spain (2). Oral delivery of vaccines would be the most practical and cost-effective means of vaccinating wildlife, and oral vaccines have been successfully used to protect foxes against rabies (109). In a study of 20 wild boar piglets aged 3–4 months, there was no increase in PPD-B or MPB83 antigen-specific antibody levels following oral BCG vaccination for 8 weeks post-vaccination (72). However, oral vaccination with heat-inactivated *M.bovis* in wild boar has been shown to induce specific antibody responses that may be associated with protection from *M.bovis* challenge (72, 110).

### Respiratory BCG

Since TB is primarily a pulmonary infection, targeting delivery of BCG directly to the respiratory mucosa (either by intranasal, aerosol or pulmonary administration) may be physiologically and immunologically beneficial (111). Furthermore, intranasal or aerosol routes could offer practical advantages for vaccine administration and negate the risk of cross-contamination due to needle re-use, particularly in developing countries.

#### Intranasal BCG

Recent studies have demonstrated that intranasal (IN) vaccination with BCG results in superior protection of mice against challenge with *M.tb* or *M.bovis* (112, 113). Intranasally administered BCG has been shown to induce secretion of total (specific and non-specific) IgA and *M.tb*-specific IgA in the lungs in an IL-17A-dependent manner at levels significantly higher than in unvaccinated mice or those vaccinated with BCG subcutaneously. This was associated with protection against pulmonary *M.tb* challenge following IN but not subcutaneous BCG in the TB-susceptible DBA/2 mouse strain (73). In a separate study in BALB/c mice, IN BCG induced significantly higher levels of specific IgA, IgG1, and IgG2a antibodies in the nasal lavage compared with subcutaneously vaccinated or unvaccinated mice and resulted in a lower bacterial load in the lungs following *M.tb* challenge (74). However, it has been noted that extent of granuloma formation is associated with level of protection in the lungs following IN BCG vaccination, and that dosage needs balancing to avoid undue pathology (114). The use of IN vaccination in humans has raised safety concerns following association of facial nerve paralysis (Bell's palsy) with three separate IN vaccines administered with the *E.coli* heat labile toxin adjuvant (ELT) (115, 116).

#### Aerosol BCG

The aerosol route of vaccination has been explored for other respiratory pathogens including influenza and measles, in which boosting with an aerosolised vaccine evoked a stronger and more durable antibody response than injected measles vaccine and was effective and well-tolerated (117, 118). BCG administered by aerosol has been demonstrated to induce a greater degree of protection against challenge with virulent *M.tb* than subcutaneous BCG vaccination in Rhesus macaques and guinea pigs (119, 120). In a separate NHP study, median anti-PPD IgG titers in the serum increased following aerosol BCG vaccination at both a standard and low-dose relative to baseline (75). Although aerosol vaccination with BCG has been described in humans, the antibody response was not measured (121). Interestingly, anti-vector antibodies to the TB vaccine candidate MVA85A were reported in the serum after ID but not aerosol administration in humans, suggesting that aerosol vaccination may overcome detrimental pre-existing humoral immunity against the vaccine vector (122).

#### Pulmonary BCG

The intratracheal (IT) and endobronchial routes may be favored for preclinical respiratory BCG studies as they allow for accurate delivery of defined vaccine doses. Although IT BCG vaccination in mice has been shown to confer superior protection to the SC route, to our knowledge there are no descriptions of the humoral response (123). In cattle, Buddle et al. reported superior protection to *M.bovis* challenge following BCG vaccination by the IT route compared with the SC route or with ID vaccination using heat-killed *M.vaccae*. However, the proportions of animals showing positive responses in an antibody ELISA against *M.bovis* culture filtrate did not differ between groups (85).

Dijkman et al. conducted a controlled NHP study in which BCG vaccination was administered by the standard ID route or by endobronchial installation into the left lower lung lobe (BCG.muc) (76). There was a significant but modest increase in PPD-specific pan-immunoglobulin in the serum following BCG vaccination by both routes compared with unvaccinated controls. IgA was significantly increased by more than 1 log in the BAL (but not the serum) at 8 weeks following BCG.muc compared with unvaccinated controls or animals receiving ID BCG vaccination. BCG.muc was able to prevent infection with repeated low-dose *M.tb* challenge (RLD), as evidenced by lack of IGRA conversion or TB-associated pathology in some of the animals. BCG.muc also elicited superior protection from RLD compared with ID BCG (~1.5 vs. ~1 log reduction in primary lung lobe CFU and ~3 vs. ~1 log reduction in BAL CFU), and importantly IgA was identified as a correlate of local protective immunity (76). IgA is the most abundantly produced natural antibody isotype in mucosal tissue, and may play an important role in the host's early defense against pathogens invading the respiratory tract (124). Indeed, it has been shown that IgA can prevent the adsorption of bacteria at the mucosal epithelium (125–127) and block the entrance of mycobacteria into the lungs (128). While endobronchial installation may not be a deployable vaccine strategy in humans, the protective signal offers a valuable opportunity to identify immune correlates of protection to aid in development of improved TB vaccines.

#### Intravenous BCG

There has been a resurgence of interest in intravenous (IV) BCG given the recent NHP study demonstrating superior protection against virulent *M.tb* challenge following vaccination by this route compared with ID or ID with an intratracheal mucosal boost (ID+IT) (129). This finding supports several reports published in the 1970s indicating improved protection conferred by the IV route of administration (119, 130–132). One early NHP study comparing BCG administered intracutaneously (IC), IV or by aerosol inhalation detected precipitating antibodies during the post-vaccination period only in the IV group; this increase was associated with marked increases in serum IgG, IgA, and IgM, although corresponding levels of protection were not measured (77). IgG, IgA, and IgM PPD-specific antibody responses were also analyzed in mice receiving IV BCG vaccination and re-vaccination 1 month later. IgG and IgM responses were detected at 1 month post-primary BCG vaccination and were further boosted by re-vaccination, remaining stable until the end of the experiment. IgA responses remained low even after two BCG vaccinations (71).

## Variation in BCG Efficacy

In addition to directing improved TB vaccine design, better characterization of the overall BCG-induced immune response may aid in understanding the observed variability in vaccine efficacy. This is essential if we are to avoid a new generation of vaccines being subject to the same pitfall. Although previous exposure to non-tuberculous mycobacteria (NTM) resulting in “masking” or “blocking” of the BCG-induced immune response is the leading hypothesis for variation in vaccine efficacy, a range of other factors such as genetic or nutritional differences between host populations, environmental influences, and viral or helminth infections at time of vaccination have been implicated (5, 133, 134).

Variation in BCG strain may also play a role, with some evidence of a divergence in protective immunity conferred by different strains (135, 136). Interestingly, a comparative analysis of human B cell epitopes based on BCG genomes indicated strain-specific differences which the authors hypothesize may contribute to variability in BCG vaccine efficacy (137). In one study comparing oral immunization of mice using BCG Pasteur, Glaxo, Japanese, Russian or Prague strains, the highest IgG and IgA anti-PPD antibody responses were observed in sera of mice immunized with the Pasteur and Russian strains (71). After IV vaccination, the Japanese strain induced only very low levels of anti-PPD specific antibodies compared with the other strains but no correlation was observed between induction of antibody and stimulation of protective immunity as measured by ability to eliminate rBCG expressing β-glacatosidase (71).

Using the Moreau strain, Petricevich et al. investigated whether differences in methodology for vaccine manufacture influenced the capacity of BCG to induce a humoral immune response in mice. They found that BCG bacilli cultured in Sauton-asparagine medium multiplied more and induced a stronger humoral immune response (with higher titers of BCG-specific antibody and a higher number of antibody-producing spleen cells) compared with bacilli grown in Sauton-starch/bacto-peptone-enriched medium (138). The mycobacterial capsule is enriched in polysaccharides and lipids and includes some immunologically active secreted proteins (139). Prados-Rosales et al. used specific antibodies to the major capsule polysaccharides, AM and α-glucan, to demonstrate that growing BCG in the presence of detergent strips the capsule. While un-encapsulated BCG induced predominantly IgM responses following vaccination, encapsulated BCG grown in the absence of detergent was better at eliciting higher titers of class-switched antibody. Encapsulated BCG was also more effective at generating IFN-γ and polyfunctional T cell responses and together this enhanced immunogenicity was associated with a lower bacterial burden following *M.tb* challenge (140).

## Off-Target Humoral Immunomodulatory Effects of BCG Vaccination

### Infectious Diseases and Heterologous Vaccines

There is a growing body of literature proposing non-specific benefits of BCG vaccination including a reduction in all-cause infant mortality and protection against unrelated infectious diseases (141–144). Although IV BCG has been reported to protect mice against challenge with *Babesia* and *Plasmodium* spp., this is not thought to be associated with antibody specific for surface antigens of the parasites (145). Furthermore, resistance of BCG-infected mice to superinfection with *Salmonella typhimurium* is not associated with a greater or more rapid antibody response (146). It is thought that protection beyond the target pathogen could rather be promoted by heterologous lymphocyte activation or innate immune memory (147). Interestingly, in a recent trial in Denmark, BCG vaccination was associated with a reduced rate of infant hospitalization for infection, but only in infants of mothers who were BCG-vaccinated (148). This observation was supported by a similar finding in Guinea-Bissau, where the reduction in all-cause mortality was 66% (95% CI, 33–83%) if the mother also had a BCG scar (149). Vaccinating against measles in the presence of maternal antibody is associated with reduced child mortality (150), and it is possible that a similar mechanism acts to enhance the non-specific effects of BCG vaccination. Indeed, studies have demonstrated that maternal BCG vaccination modifies the immunological response to BCG in the infant (151, 152). In the BCG re-vaccination trial described in the section BCG Re-vaccination, a decrease in unrelated respiratory tract infections was observed in BCG re-vaccinated individuals compared with the H4:IC31 vaccinated or placebo groups (102, 153). Although the immune mechanism remains unclear, BCG re-vaccination, like maternal priming, represents a situation in which the presence of (possibly antibody-related) pre-existing immunity enhances non-specific effects.

There is some evidence that BCG vaccination enhances the humoral immune response to other unrelated childhood vaccinations, essentially acting as an adjuvant. Ota et al. reported that infants vaccinated at birth with BCG had, upon vaccination against hepatitis, significantly higher levels of IgG against hepatitis B vaccine antigens. Furthermore, BCG vaccination given at the time of oral polio vaccine boosting improved the antibody response to polio, indicating an effect of BCG at the systemic level (154). An Australian study of 56 BCG-vaccinated and 52 non-BCG vaccinated infants found that BCG vaccination was associated with significantly higher IgG titers against pneumococcal capsular polysaccharide antigens, *H.influenzae* type b polysaccharide and tetanus toxoid following routine immunisations later in infancy (155). Conversely, in a study of 300 children in a high-income setting, no overall effect of neonatal BCG vaccination was observed on antibody levels induced by the routine vaccinations DiTeKiPol/Act-Hib and Prevnar 13. However, a possible enhancing effect of BCG on antibodies against *B. pertussis* and all pneumococcal serotypes was identified when stratifying by age of randomization to BCG vaccination or no BCG vaccination suggesting that the timing of BCG vaccination may be important (156).

### Atopic Disorders and IgE

The hygiene hypothesis proposes that the increasing occurrence of allergies in the developed world may be associated with reduced incidence of microbial infections, including TB, which would otherwise bias the developing immune system toward a Th1 response. The resulting Th1-Th2 imbalance with an increase in Th2 cells favors IgE antibody production and the establishment of allergic reactivity (157). In such a situation, it is plausible that BCG early in life could provide Th1 stimulation that mimics mycobacterial infection and reduces the incidence of atopy. Indeed, positive tuberculin responses were associated with lower serum IgE levels, cytokine profiles biased toward a Th1 type and lower incidence of asthma in a retrospective study of over 800 Japanese schoolchildren (158). In Italian volunteers with allergic rhinitis, there was a significant decrease in total and allergen-specific IgE levels following BCG vaccination (159). A further study indicated that BCG vaccination was associated with downregulation of spontaneous and stimulated *in vitro* IgE secretion from PBMC of atopic children with asthma (160). Atopy (defined as skin test reactivity to *Dermatophagoides pteronyssinus, D. farinae* or cockroach) was reduced in African children given BCG vaccination early in infancy, although IgE levels were not measured in this study (161). That BCG vaccination is associated with a reduction in allergen-specific antibodies and a beneficial effect on atopic disorders is supported by several preclinical studies (162–165).

However, others report no marked effect of BCG vaccination on IgE levels or risk of development or symptoms of atopic disease (166–171). While there are several studies describing repeated administration of inactivated BCG for the management of asthma (172–174), a randomized double-blind placebo-controlled study in adults with moderately severe asthma and house dust mite allergy showed no efficacy of four injections with heat-inactivated BCG and no reduction in IgE compared with the placebo group (175). Differences in outcome may be related to time of, or since, BCG vaccination (176, 177) or host genetic factors (178, 179). Safety concerns have been raised regarding the use of BCG as a tool for the prevention and therapy of allergic airway disease, as Ahrens et al. noted that BCG-induced suppression of Th2-type allergic airway inflammation in the OVA-sensitized mouse also stimulated Th1-associated neutrophilic airway inflammation (180).

### Autoantibodies

Autoantibodies have been found at high frequency in the blood of patients infected with mycobacteria (181), suggesting cross-reactivity between mycobacterial and host antigens. A recent study of sera obtained from healthy adults post-BCG vaccination reported IgG recognition of host peptides that peaked at 8 weeks post-vaccination and diminished over time (182). IgG responses primarily demonstrated increased reactivity to ion transporters, cytokine receptors, ribonucleoprotein and enzymes. The authors suggest that the modification of the host immunological and non-immunological landscape in this way may influence disease-specific immune responses previously reported in TB, autoimmune disease and cancer (182). Indeed, autoantibodies associated with autoimmune diseases such as systemic lupus erythematosus (SLE) have been shown to recognize mycobacterial antigens (183, 184). In non-obese diabetic (NOD) mice, heat-killed BCG was shown to prevent diabetes but precipitated a systemic lupus erythematous (SLE)-like syndrome associated with increased titers of antinuclear autoantibodies (185).

Insulin-dependent diabetes mellitus, or type 1 diabetes (T1D), is an autoimmune condition in which insulin-producing pancreatic islet cells undergo immune-mediated destruction. BCG vaccination has been reported to prevent the onset of T1D in mice and result in clinical remission or a long-term reduction in blood sugar levels in T1D patients (186–189). T1D is a predominantly T cell-mediated disease, and the mechanism of action of BCG in this context is thought to be through induction of tumor necrosis factor (TNF) resulting in selective death of autoreactive T cells and expansion of beneficial regulatory T cells that restore immune balance (190). Autoantibodies may also play a role and BCG vaccination has been associated with a significant reduction of autoantibodies to the 65-kDa isoform of glutamate decarboxylase (GAD65) and islet tyrosine phosphatase (I-A2) in southern Indian diabetic patients (191). However, a separate prospective study of German infants born to parents with T1D found no evidence that BCG vaccination reduced GAD65, I-A2 or insulin autoantibody levels by age 2 or 5 years, and suggested that neonatal BCG vaccination may in fact accelerate progression from autoimmunity to diabetes in autoantibody-positive children (192). Furthermore, BCG vaccination has been linked to an increased risk of developing high levels of anti-IA-2 autoantibodies, although the authors note that the number of vaccinated children was relatively small and not representative of the general population (193). Outcomes have been shown to differ widely from individual to individual (194).

Multiple sclerosis (MS) is an autoimmune-mediated inflammatory disease affecting the central nervous system. It has been postulated that BCG vaccination may be beneficial in suppressing autoimmune responses in the animal model of MS (experimental autoimmune encephalomyelitis) and human MS (195, 196), and conversely that it could initiate autoimmunity through molecular mimicry (197). Interestingly, a seroprevalence study measuring antibodies against the host encephalitogenic myelin oligodendrocyte glycoprotein (MOG)_35−55_ epitope and two mycobacterial peptides sharing sequence homology showed a significant difference between MS patients and healthy controls in levels of antibody positivity for MOG_35−55_ but not for the BCG-derived homologous peptides (198). A possible role for antibody responses to mycobacterial hsp65 (and equivalent human or bacterial proteins) has been proposed in rheumatoid arthritis (RA), although findings are conflicting (199–201). Increased levels of anti-BCG antibodies have also been reported in RA and SLE patients compared with healthy controls (202), and BCG therapy for cancer has been associated with systemic autoimmune phenomena such as inflammatory arthritis or immune-related toxicity, likely through molecular mimicry (203–205).

### Cancer Immunotherapy

The relationship between mycobacteria and cancer was first recognized almost a century ago, with animal studies demonstrating that BCG-infected mice were resistant to transplantation of tumor cells leading to the discovery of Tumor Necrosis Factor (TNF) (206–208). Numerous attempts were consequently made to apply BCG as a therapy for various cancers including leukemia and melanoma; the efficacy of BCG therapy for bladder cancer has since been confirmed and it is now the standard of care for high-risk non-muscle-invasive disease (209). It is generally considered that CD4^+^ and CD8^+^ T cells, NK cells and granulocytes are the main players in mediating the immunotherapeutic effect of BCG in this context, but important humoral factors have been identified and it is possible that antibodies contribute to the immune activation observed (210). While some early studies in melanoma and bladder cancer patients reported an increase in BCG-specific antibody following therapy which was tentatively associated with reduced rates of recurrence (211–213), others did not confirm these findings (214, 215). Interestingly, parenteral exposure to BCG prior to intravesical treatment has been associated with improved response to therapy, and patients with sustained pre-existing immunity to BCG showed significant improvement in recurrence-free survival (216). This is in line with the observations regarding the beneficial effects of maternal priming and BCG re-vaccination on non-specific effects of BCG described in the section Infectious Diseases and Heterologous Vaccines.

Antibodies raised against mycobacterial antigens with a high degree of homology to proteins found on the surface of tumor cells may be of direct relevance to clinical outcome. Early work on cross-reactivity demonstrated that guinea pigs immunized with BCG developed antibody in their sera to the transplantable guinea pig hepatoma line 10 (but not line 1) (217). The mycobacterial heat shock protein hsp65 induces a strong cellular and humoral immune response, and hsp65 from BCG has been shown to enhance recognition of tumor-associated antigens (218). It has been reported that titers of IgG against hsp65 and the native protein P64 in bladder cancer patients increase after BCG intravesical therapy, but that increased antibody responses against P64 may be associated with a higher rate of tumor recurrence (215, 219). In a murine model of transplantable methylcholanthrene-induced fibrosarcoma, BCG treatment of tumor-bearing mice stimulated formation of antibodies against tumor-specific antigens but these antibodies enhanced tumor growth (220). A summary of the off-target humoral immunomodulatory effects (both beneficial and detrimental) of BCG vaccination is provided in Table 3.

**Table 3:**
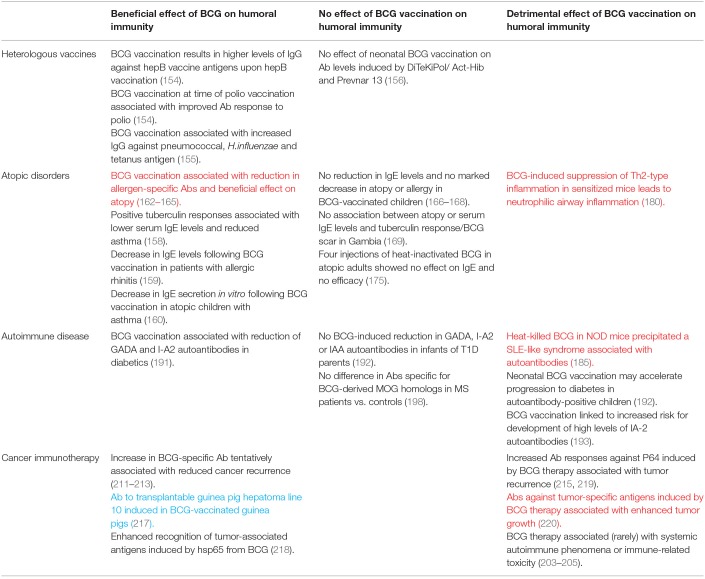
Summary of evidence for off-target humoral immunomodulatory effects of BCG vaccination discussed in this review.

*Human studies are indicated by black text, murine studies are indicated by red text and studies in any other species are indicated by light blue text*.

## Conclusions

Until relatively recently, a simplistic paradigm has prevailed of a mutually-exclusive immunological duality with the cellular and humoral arms of immunity conferring protection against intracellular and extracellular pathogens respectively. However, this was challenged by Casadevall and others who pointed to a role for humoral immunity in preventing infection with some intracellular pathogens (221). It is now recognized that there is extensive synergy between the two arms of immunity with, for example, B cells modulating the T cell response via cytokine production and participating in T cell priming through antigen capture and presentation (40, 222). A dual requirement for both cell-mediated and humoral immunity in the development of an effective response to both intracellular and extracellular pathogens is increasingly recognized. Furthermore, with accumulating evidence supporting a contribution of trained innate immunity to protection (19), the key to a more successful TB vaccine may lie in the stimulation of the innate immune system and harnessing of humoral immunity in concert with a potent cell-mediated response.

In light of this, together with recent evidence supporting a role for antibodies in immunity against TB and questioning the sufficiency of the cell-mediated response alone to confer protection (15, 16, 26–28), understanding humoral immunity in TB has become increasingly pertinent. As the only vaccine currently available against TB, and offering partial protection against the transmissible pulmonary form of disease, BCG offers an opportunity to identify and characterize the protective components of vaccine-induced humoral immunity. Such insights may aid in the design of new more efficacious TB and bTB vaccine candidates, especially when related to functional properties *in vitro* and protection from mycobacterial challenge or natural infection *in vivo*. Alternatively, given the recent findings regarding the non-specific benefits of BCG (141–144), the influence of maternal priming (148–152) and the efficacy of BCG re-vaccination in reducing sustained infection (102), control of the TB epidemic may in fact be achieved through wiser use of the BCG vaccine itself.

However, the humoral immune response to BCG vaccination remains poorly defined. Evidence for the induction of specific antibody responses is variable and the relevance to protection is unclear, as summarized in Table 2. Many of the cited studies suffer from small sample sizes and unspecified/different sampling time-points or duration of follow-up. Outcomes may also be influenced by assay methods, dose and strain of BCG vaccine, species/strain of animal or population of humans and baseline characteristics such as mycobacterial exposure, co-infections or genetic factors. Age at time of vaccination is also important; the presence of maternal antibody in early infancy may complicate studies of humoral immunity to neonatal vaccination and durability of induced antibody responses is more limited than at older ages. In particular, when literature is consolidated, it is apparent that BCG-induced antibody is observed more consistently in humans than cattle. However, findings in cattle, at least in the earlier studies, may be confounded by the use of high doses of BCG which are now known to be less effective than lower doses, and that studies were often performed in regions with very high prevalence of *M.bovis* or in neonatal calves with immature immune systems and presence of maternal antibody (2).

Recently, large randomized controlled trials using standardized assays have more consistently observed induction of antibody following BCG vaccination, with convincing evidence for a functional role of LAM- or AM-specific IgG *in vitro* (53, 54). It is clear from several studies that BCG induces antibody directed against LAM and AM, and that vaccine constructs based on these antigens can be protective in mice (84). However, other antigen targets are not well-defined and protective epitopes may not necessarily be on the cell surface, be immunodominant or induce the highest antibody titers. This area warrants further investigation, including consideration of other measures of antibody efficacy such as avidity and affinity which may associate more strongly with protection than titer alone.

Inability to observe a correlation with protection does not rule out antibody responses as a contributing factor; humoral immunity may be necessary but not sufficient for protection, or may be relevant for some hosts but not others. Furthermore, the overwhelming majority of studies investigating anti-mycobacterial humoral responses have sampled the periphery, which may not reflect the situation at the site of infection. As different routes of vaccination are explored, particularly targeting the respiratory mucosa, the induction of localized humoral immunity may become increasingly relevant. IgA, the predominant antibody isotype present in the mucosal tissue, is widely considered to be involved in defense against viral and bacterial infections at these sites, and it has been demonstrated that IgA-deficient mice have increased susceptibility to IN mycobacterial infection (223). Furthermore, Balu et al. reported protection against *M.tb* in mice using an immunotherapeutic human IgA monoclonal antibody (224).

Better characterization of the BCG-induced immune response may also aid in understanding the observed variability in vaccine efficacy, which is essential if we are to avoid a new generation of TB vaccines being subject to the same pitfall as BCG. Differences in the humoral immune response observed between BCG strains and vaccine manufacture methods may be one contributing factor. It would also be of interest to compare BCG-induced antibody responses between populations where BCG varies in efficacy and determine potential associations with NTM exposure. A study by Sebina et al. found no differences in mycobacteria-specific antibody levels between volunteers with or without travel history to *M.tb* endemic areas, but indicated that some unvaccinated uninfected individuals possess cross-reactive antibodies and memory B cells likely induced by exposure to NTMs or other pathogens (44).

It has long been recognized that BCG has complex and diverse immunomodulatory influences ranging from effects on autoimmune disease to atopic disorders and cancer. Such off-target effects are relevant in the context of vaccination as a TB vaccine candidate designed to replace BCG should ideally be non-inferior in non-specific as well as specific efficacy. Furthermore, as novel candidates in the current pipeline include both homologous and heterologous BCG prime-boost regimens, understanding the mechanism of non-specific immunity opens the possibility of boosting such responses to the host advantage. However, study findings are often divergent, and subject to the same confounders as those of specific responses to BCG such as time of, or since, vaccination and additional external influences on the immune system. While it is clear that the effect of BCG vaccination on IgE antibody responses and atopic disorders is beneficial in at least some populations, heterologous humoral immune responses in other contexts may represent a double-edged sword leading to autoimmunity through molecular mimicry.

In conclusion, as we begin to appreciate a role for the humoral immune response in protection from TB, further studies should be aimed at elucidating the nature and relevance of the humoral immune response to BCG vaccination and how this may be harnessed to better design novel candidate vaccines for TB and bTB.

## Author Contributions

RT wrote the first draft of the manuscript. BV-R, HMV, and HM wrote sections of the manuscript. All authors contributed to manuscript revision, read and approved the submitted version.

### Conflict of Interest Statement

The authors declare that the research was conducted in the absence of any commercial or financial relationships that could be construed as a potential conflict of interest.
